# Editorial: 15 years of frontiers in cellular neuroscience: myelination and remyelination processes

**DOI:** 10.3389/fncel.2024.1463579

**Published:** 2024-08-09

**Authors:** Qingchao Qiu, Bo Hu

**Affiliations:** ^1^Department of Veterans Affairs, Michael E. DeBakey VA Medical Center, Houston, TX, United States; ^2^Department of Neurology, Houston Methodist Research Institute, Houston, TX, United States

**Keywords:** myelination, remyelination, demyelination, glial cells, oligodendrocytes, Schwann cells, neuron, axon

## Introduction

**Figure 1:**
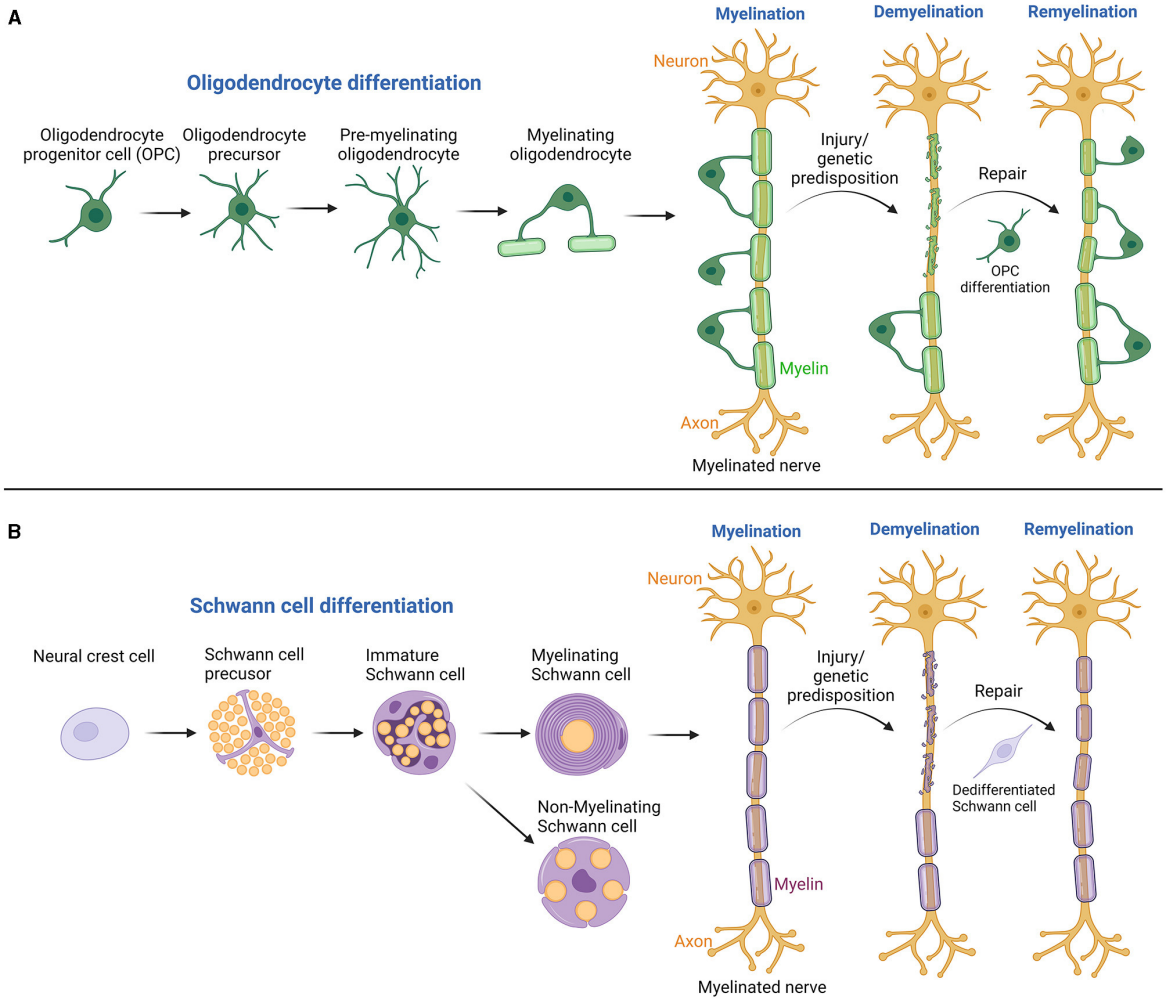
Illustrates the processes of myelination and remyelination in both the CNS and PNS. **(A)** In the CNS, the process starts with oligodendrocyte progenitor cells and progresses through several stages to produce myelinating oligodendrocytes. Following demyelinating injuries, these progenitor cells migrate, proliferate, and differentiate at the injury site, leading to remyelination by forming new myelin sheaths. **(B)** In the PNS, Schwann cells evolve from neural crest cells through several developmental stages, culminating in mature myelinating or non-myelinating Schwann cells. These mature cells are pivotal in forming the myelin sheath, which is essential for insulating axons, thereby enhancing the speed and efficiency of electrical signal transmission across nerve fibers. Damage to these cells can lead to demyelination, with adjacent Schwann cells undergoing dedifferentiation and redifferentiation to support nerve regeneration. Created with BioRender.com.

Myelin, the insulating sheath around nerve axons, plays a critical role in the conduction of electrical signals within the nervous system (Nave, 2010). Comprising nearly half of the brain's white matter and even more in peripheral myelinated axons, myelin is essential for proper nervous system development and maintenance. Myelin sheaths are lipid-rich substances produced by oligodendrocytes in the central nervous system (CNS) and Schwann cells (SCs) in the peripheral nervous system (PNS) (Figure 1) (Balakrishnan et al., 2020; Yu et al., 2023). One oligodendrocyte in the CNS can insulate several axons, unlike SCs in the PNS, which wrap around only a single axon. Decades of research, propelled by cutting-edge technologies, have significantly advanced our knowledge of myelin's structure, functions, and the dynamic processes of myelination and remyelination. Myelination, regulated by a precise genetic program, is essential for developmental neurobiology and ongoing neuronal function and integrity (Sock and Wegner, 2019). Disruptions in myelination, seen in conditions like multiple sclerosis, Guillain-Barre syndrome, and Charcot-Marie-Tooth disease, can result in severe neurological deficits (Mehndiratta and Gulati, 2014). However, the nervous system can repair itself through remyelination, a regenerative process where damaged myelin sheaths are repaired or replaced. This process is carried out by glial progenitor cells in the CNS and residual SCs in the PNS (Momenzadeh and Jami, 2021). Understanding these mechanisms is key to developing treatments for a wide array of neurological disorders, highlighting the importance of myelination and remyelination in neurobiology. This editorial synthesizes findings from two reviews and two research articles, shedding light on the latest advancements in neuroimaging and cellular biology that enhance our understanding of myelin dynamics in both healthy and diseased states of the nervous system.

## Articles in this Research Topic

Modern neuroimaging techniques provide crucial insights into myelin dynamics in the human brain, both under normal and pathological conditions (Laule et al., 2007). The review by Kujawa et al. presents new magnetic resonance imaging (MRI) techniques and biophysical models to map myelin *in vivo*, highlighting the potential of physical exercise to influence myelination and remyelination in the human brain. It details current research, including four cross-sectional studies, four longitudinal studies, and a case report, showcasing the beneficial effects of an active lifestyle on myelin content across all ages. The findings suggest that intensive aerobic exercise can induce myelin expansion, underscoring the importance of exercise in managing demyelination in aging and neurodegenerative conditions. This review advocates for further research to determine the most beneficial exercise intensities for neurological health, making it an invaluable asset for clinical and research applications.

The remarkable adaptability of SCs is crucial after nerve damage or in cases of demyelinating neuropathies, where they first dedifferentiate and then redifferentiate to aid in nerve regeneration and recovery (Boerboom et al., 2017). The review article by Zhang et al. discusses the critical issue of delayed peripheral nerve injury (PNI) repair in elderly patients, focusing on the role of aging SCs. As the primary facilitators of nerve repair, SCs orchestrate various reparative functions, including demyelination, secretion of neurotrophic factors, and axon remyelination. This review highlights how structural and functional changes in aged SCs contribute to diminished nerve repair capabilities that result in chronic pain, muscle atrophy, and severe disability. Exploring these age-related alterations emphasizes the urgent need for further research into SC biology to potentially enhance therapeutic strategies for PNI in the elderly.

The specific localization of proteins within the nervous system's various cells is crucial for their functionality, impacting nerve development and maintenance (Sock and Wegner, 2019). The study by Fazal et al. focuses on the distribution and functionality of SARM1 in myelinating glia cells, addressing whether its dysfunction could impact neuropathology or interfere with myelination therapies. The study reveals that while SARM1 mRNA and protein are present in oligodendrocytes, with activation leading to cell death, it is notably absent or non-functional in peripheral glia such as SCs and satellite glia. This suggests that therapeutic strategies targeting SARM1 to preserve axons in nervous system diseases are unlikely to affect myelination negatively. The findings also underscore that SARM1 is not necessary for the initiation or maintenance of myelination in both the central and peripheral nervous systems. This research crucially informs the development of SARM1 inhibitors as potential treatments for neurological disorders, providing a clearer path for targeting this protein without harming myelin integrity.

Demyelinating diseases cause severe long-term neurological damage. Promoting remyelination can restore nerve function and prevent further neuronal loss and clinical disability, driving research into drugs that could enhance remyelination for therapeutic use (Harlow et al., 2015). Cisneros-Mejorado et al. explore the potential of β-carbolines to enhance remyelination in a rat model of demyelination in the inferior cerebellar peduncle (DRICP model). Employing the DRICP model, the authors induced demyelination using ethidium bromide, confirming the damage histologically and assessing it with diffusion-weighted MRI. The study evaluated the remyelinating effects of three β-carbolines that modulate the GABAA receptor in oligodendrocytes. Notably, the N-butyl-β-carboline-3-carboxylate (β-CCB) and ethyl 9H-pyrido [3,4-b]indole-3-carboxylate (β-CCE) demonstrated significant efficacy in promoting remyelination as evidenced by improved dMRI metrics and increased myelin content histologically. These findings suggest that specific β-carbolines could be promising in therapeutic strategies targeting white matter recovery.

## Concluding remarks

The Research Topic of articles reviewed herein not only enrich our understanding of myelin dynamics in both normal and pathological states but spotlight the intrinsic capability of the nervous system to adapt and repair itself. From enhancing our grasp of neuroimaging techniques to unraveling the molecular intricacies of myelination and remyelination processes, these studies contribute to neuroscientific progress; in addition, they emphasize the potential of targeted physical and pharmacological interventions to mitigate or reverse the effects of demyelinating conditions. As research continues to evolve, these insights hold the promise of improving diagnostic, therapeutic, and preventive strategies in neurology, thereby bettering patient outcomes in the face of debilitating neurological diseases. This underscores the critical importance of ongoing research in myelin biology as a cornerstone for advancing our approach to neurological health and disease management.

## Author contributions

QQ: Methodology, Software, Writing – original draft, Writing – review & editing. BH: Conceptualization, Software, Supervision, Writing – original draft, Writing – review & editing.
